# Supplementary material for: Enrichment of human IgA-coated bacterial vesicles in ulcerative colitis as a driver of inflammation
Source: Nat Commun. 2025 Apr 29;16:3995. doi: 10.1038/s41467-025-59354-5 (PMC12041585; doi:10.1038/s41467-025-59354-5)
Supplement: Supplementary file 1 — Supplementary Information [file 41467_2025_59354_MOESM1_ESM.pdf]

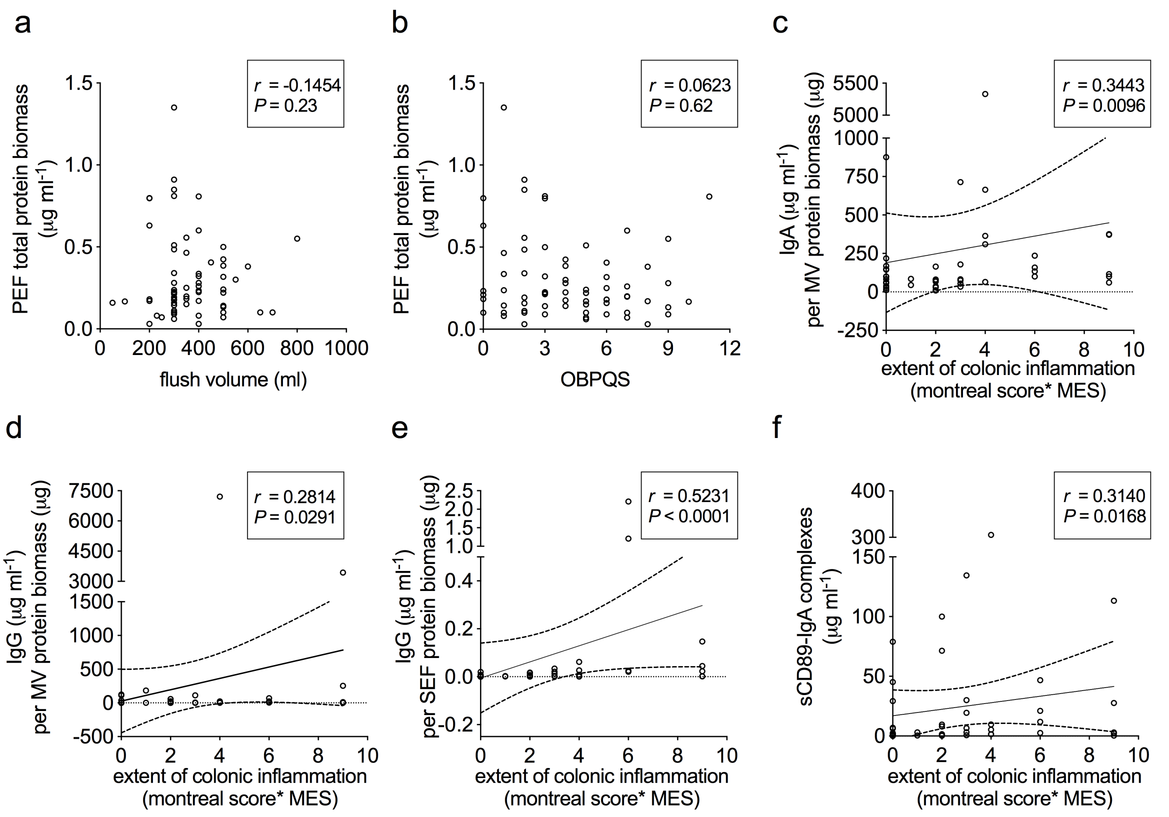

Supplementary Figure 1: Correlation summary soluble and pelletable fractions derived from non-IBD and UC patients.

a, Total protein biomass of the pelletable effector fractions (PEF) does not correlate with the flush volume during colonoscopy. n=66, $r_{Spearman}$ = -0.1454; *P* =0.23 (two-sided),

b, Total protein biomass of the pelletable effector fractions (PEF) does not correlate with the patient bowel preparation prior to colonoscopy determined by the Ottawa Bowel Preparation Quality Scale (OBPQS). n=70, $r_{Spearman}$ = 0.0623; *P* =0.62 (two-sided)

c, IgA levels of EV fractions from UC patients (n=46) correlates with colonic inflammation. $r_{Spearman}$ = 0.3443; *P* =0.0096 (two-sided)

d, IgG levels of EV fractions from UC patients (n=46) correlates with colonic inflammation. $r_{Spearman}$ = 0.2814; *P* =0.0291 (two-sided)

e, IgG levels of soluble effector fractions (SEF) from UC patients (n=46) correlates with colonic inflammation. $r_{Spearman}$ = 0.5231; *P* <0.0001 (two-sided)

f, Amount of sCD89-IgA complexes in EV fractions from UC patients (n=46) correlates with colonic inflammation. $r_{Spearman}$ = 0.3140; *P* =0.0168 (two-sided)

c - f, The colonic inflammation index integrates the macroscopic inflammatory activity and the length of inflamed colonic tissue and is calculated as follows: Mayo endoscopic subscore (MES; 0-3) x Montreal score (1-3). The EMS grades inflammation seen macroscopically on colonoscopy in UC patients in no inflammation (0), mild (1), moderate (2) or severe (3)

inflammation [1-3]. The Montreal classification defines the maximal extent of colonic inflammation in patients with ulcerative colitis, into proctitis (1), left sided colitis (2) or extensive colitis and pancolitis (3) [4].

a - f, Spearman correlation coefficient between two measurements and the corresponding *P* value (two-sided) are shown. Source data are provided as a Source Data file.

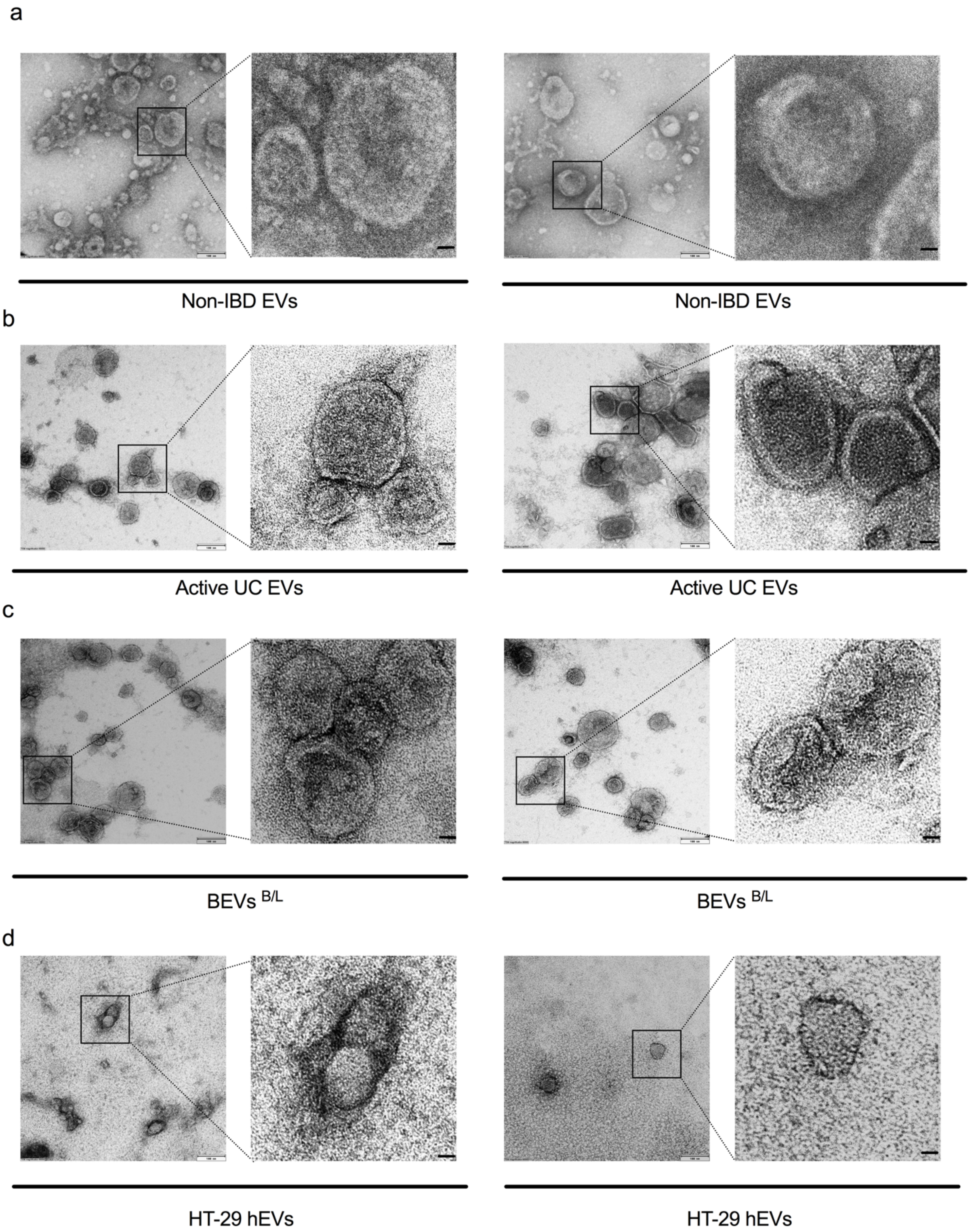

Supplementary Figure 2: Visualization of extracellular vesicles (EVs) by transmission electron microscopy (TEM).

Shown are representative TEM images visualizing EVs with typical morphology in two randomly chosen PEF samples from the non-IBD control group (panel a, non-IBD EVs), of two randomly chosen PEF samples from the active UC patients (panel b, active UC EVs), of two preparations of bacterial-derived extracellular vesicles from *L. acidophilus* and *B. fragilis*

(BEVs$^{B/L}$) (panel c) and two preparations of host-derived extracellular vesicles from the intestinal epithelial cell line HT-29 (hEVs, panel d). Panels on the right show magnified regions of the corresponding overview image (left) highlighted by a red rectangle Samples were negatively stained with uranyl acetate. Scale bars, 100 nm for overview images and 10 nm for the magnified sections. The experiments were repeated at least 3 times with a similar result.

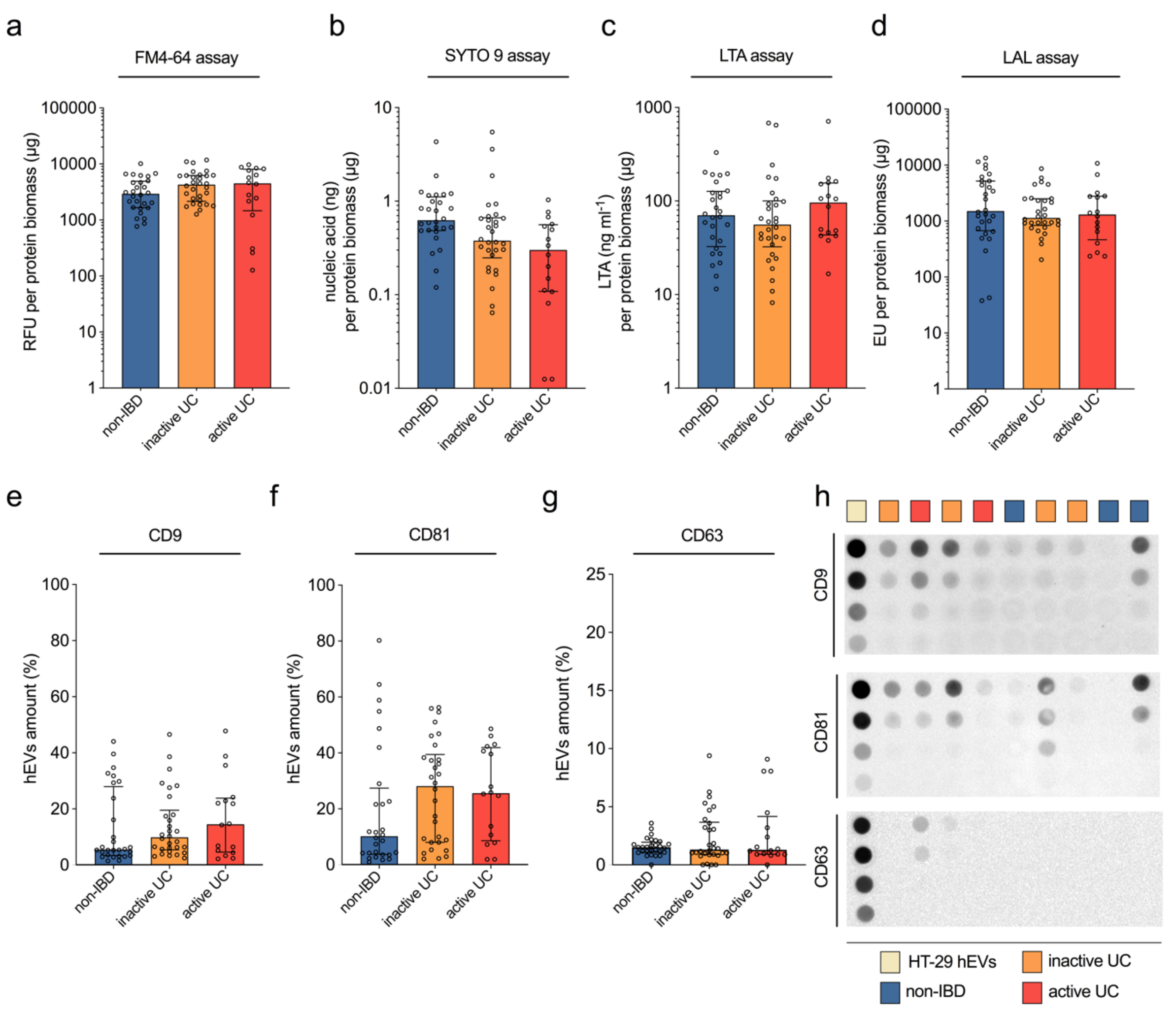

Supplementary Figure 3: Characterization of the pelletable fraction composition.

a, Lipid content quantification in pelletable effector fractions determined by the FM™ 4-64 assay. Relative fluorescence units (RFU) were measured in 1 µg total protein biomass of the respective pelletable fraction.

b, Nucleic acid content quantification in pelletable effector fractions determined by SYTO™ 9 staining. Indicated nucleic acid amounts were quantified in 1 µg total protein biomass of the respective pelletable fraction.

c, LTA amount in pelletable effector fractions determined by ELISA. Indicated LTA amounts were quantified in 1 µg total protein biomass of the respective pelletable fraction.

d, LPS activity in pelletable effector fractions determined by LAL assay. Indicated LPS activity in EU per ml were quantified in 1 µg total protein biomass of the respective pelletable fraction.

e, f, g, Shown is the percentage of hEVs in pelletable effector fractions in relation to a known amount of hEVs derived from intestinal epithelial HT-29 cells based on CD9 (e), CD81 (f) or CD63 (g) detection. Calculation was based on dot blot analyses detecting CD9, CD81 or CD63

signal intensities using hEVs isolated from HT-29 cells as standard. Representative examples for dot blots are provided in panel h. Percentage is relative to the total protein biomass.

h, Representative examples for dot blots detecting CD9, CD81 or CD63 in hEVs derived from intestinal epithelial HT-29 cells and randomly chosen pelletable effector fractions comprising non-IBD (blue, n=3), inactive UC (yellow, n=3) and active UC (red, n=2). On each dot blot four 2-fold dilutions from each sample arranged in columns (from top to bottom) were spotted onto the nitrocellulose membrane. Spotted sample amounts started at 1 µg (protein equivalent) of HT-29 derived hEVs (serving as standard on the left column of each dot blot) and 2 µg (protein equivalent) of each individual pelletable effector fraction. The relative intensities of spots derived from the four dilutions of HT-29 hEVs were used to generate a polynomial regression curve, which was subsequently used to calculate the biomarker amount of hEVs (CD9, CD81 or CD63) present in the PEF samples given by the relative intensity of the most concentrated, non-saturated sample spot. See method section for details.

a - g, Data is presented as median ± interquartile range. sample number as follows: non-IBD (blue, n=28), inactive UC (yellow, n=30) and active UC (red, n=16). Data sets of the three groups show no significant differences for any parameter displayed, which was evaluated by Kruskal–Wallis with Dunn's test.

a – h, Source data are provided as a Source Data file.

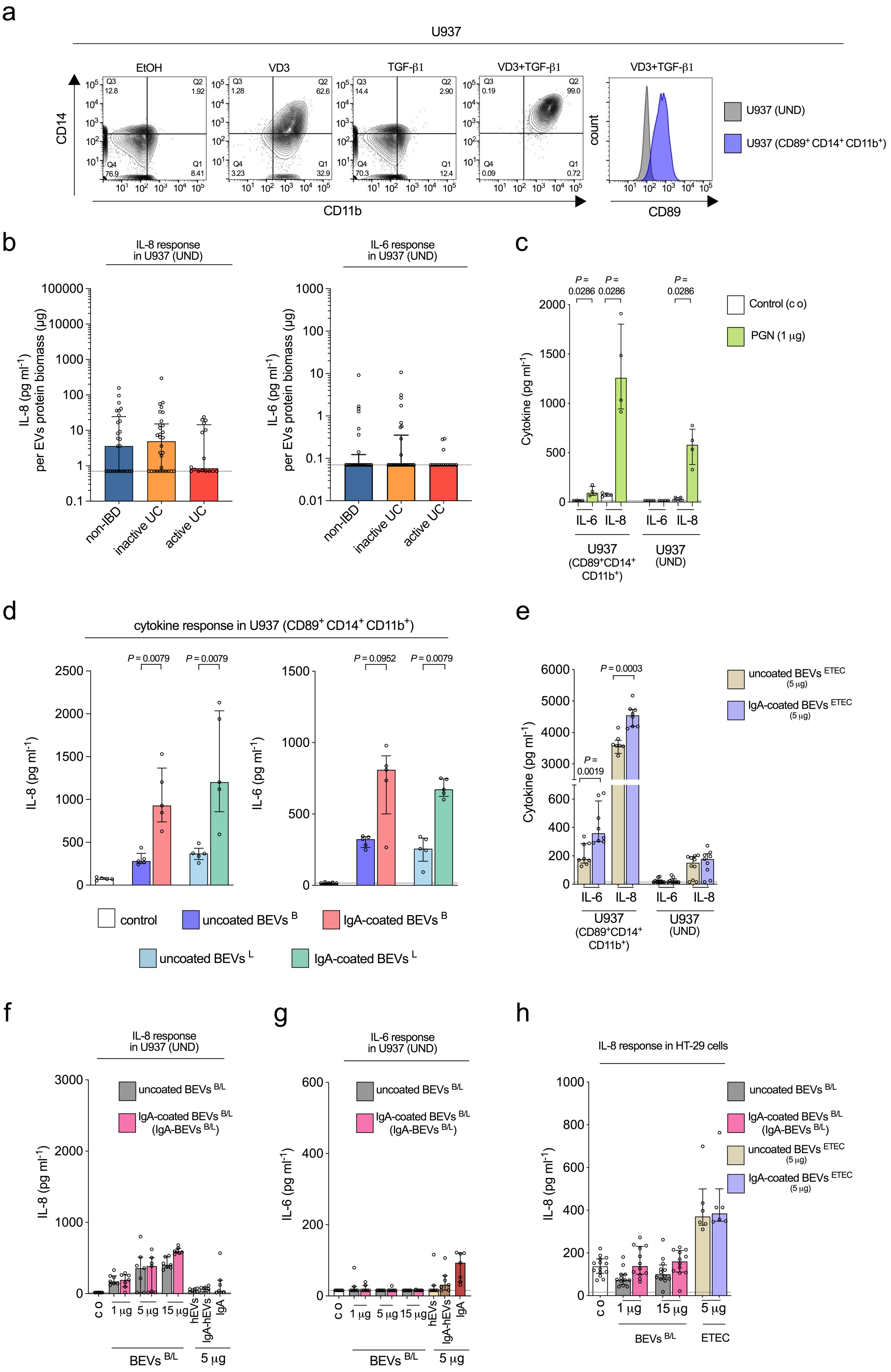
a
U937
EtOH
VD3
TGF-β1
VD3+TGF-β1
VD3+TGF-β1
CD14
CD11b
count
CD89
U937 (UND)
U937 (CD89+ CD14+ CD11b+)
b
IL-8 response in U937 (UND)
IL-6 response in U937 (UND)
IL-8 (pg ml-1) per EVs protein biomass (µg)
IL-6 (pg ml-1) per EVs protein biomass (µg)
non-IBD
inactive UC
active UC
c
Control (c o)
PGN (1 µg)
Cytokine (pg ml-1)
P = 0.0286
U937 (CD89+CD14+ CD11b+)
U937 (UND)
d
cytokine response in U937 (CD89+ CD14+ CD11b+)
IL-8 (pg ml-1)
IL-6 (pg ml-1)
P = 0.0079
P = 0.0952
control
uncoated BEVs B
IgA-coated BEVs B
uncoated BEVs L
IgA-coated BEVs L
e
P = 0.0003
P = 0.0019
uncoated BEVs ETEC (5 µg)
IgA-coated BEVs ETEC (5 µg)
f
IL-8 response in U937 (UND)
uncoated BEVs B/L
IgA-coated BEVs B/L (IgA-BEVs B/L)
hEVs
IgA-hEVs
IgA
BEVs B/L
5 µg
g
IL-6 response in U937 (UND)
h
IL-8 response in HT-29 cells
uncoated BEVs ETEC (5 µg)
IgA-coated BEVs ETEC (5 µg)
ETEC

Supplementary Figure 4: Combined stimulation of U937 cells with vitamin D3 and TGF-β1 induces the generation of CD89⁺CD14⁺CD11b⁺ monocytes, which is required to evoke a cytokine response to IgA-immunocomplexes.

a, Flow cytometry analysis of U937 cells treated with EtOH solvent control, vitamin D3 (VD3) alone, TGF-β1 (TGF-β1) alone or with VD3 + TGF-β1 in combination for CD14 and CD11b expression as well as analysis of U937 cells treated with VD3 + TGF-β1 vs control for CD89 expression.

b, IL-8 and IL-6 response in undifferentiated (UND) U937 cells exposed to EV fractions of the non-IBD (blue, n=28), inactive UC (yellow, n=30) and active UC (red, n=16) group.

c, IL-8 and IL-6 response in $CD89^+CD14^+CD11b^+$ U937 monocytes as well as undifferentiated (UND) U937 cells exposed to 1 µg peptidoglycan (PGN) or to saline serving as mock-treated control (co). (n=4 for each data sets)

d, IL-8 and IL-6 response in $CD89^+CD14^+CD11b^+$ U937 monocytes exposed to uncoated or IgA-coated BEVs from *B. fragilis* ($BEVs^B$) and uncoated or IgA-coated BEVs from *L. acidophilus* ($BEVs^L$). Dosage of the BEVs given by total protein biomass determined by Bradford is indicated. Incubation with saline served as mock-treated control (co). (n = 5 for each data set)

e, IL-8 and IL-6 response in $CD89^+CD14^+CD11b^+$ U937 monocytes as well as undifferentiated (UND) U937 cells exposed to uncoated ($BEVs^{ETEC}$) or IgA-coated BEVs ($IgA\text{-}BEVs^{ETEC}$) from ETEC. Dosage of the effector samples given by total protein biomass determined by Bradford is indicated. n=8 for all data sets with $CD89^+CD14^+CD11b^+$ U937; n=10 for $BEVs^{ETEC}$ and n=9 for $IgA\text{-}BEVs^{ETEC}$ in undifferentiated (UND) U937 cells.

f, IL-8 response in undifferentiated (UND) U937 cells exposed to uncoated or IgA-coated BEVs from *B. fragilis* and *L. acidophilus* ($BEVs^{B/L}$), uncoated or IgA-coated hEVs or soluble IgA alone. Dosage of the effector samples given by total protein biomass determined by Bradford is indicated. Incubation with saline served as mock-treated control (co). n=8 for co; n=7 for all other data sets.

g, IL-6 response in undifferentiated (UND) U937 cells exposed to uncoated or IgA-coated BEVs from *B. fragilis* and *L. acidophilus* ($BEVs^{B/L}$), uncoated or IgA-coated hEVs or soluble IgA alone. Dosage of the effector samples given by total protein biomass determined by Bradford is indicated. Incubation with saline served as mock-treated control (co). n=7 for all data sets.

h, IL-8 response in HT-29 human intestinal epithelial cells exposed to uncoated or IgA-coated BEVs from *B. fragilis* and *L. acidophilus* ($BEVs^{B/L}$ and $IgA\text{-}BEVs^{B/L}$) or uncoated or IgA-

coated BEVs from ETEC (BEVs^{ETEC} and IgA-BEVs^{ETEC}). Dosage of the effector samples given by total protein biomass determined by Bradford is indicated. Incubation with saline served as mock-treated control (co).). n=14 for co; n=6 for BEVs^{ETEC} and IgA-BEVs^{ETEC} as well as n=12 for all other data sets.

b - h, Data is presented as median ± interquartile range. Samples with no detectable signal were set to the limit of detection, which is indicated by a dotted line. Data sets of the non-IBD, inactive UC and active UC group show no significant differences for any parameter displayed, which was evaluated by Kruskal–Wallis with Dunn's test; $^*P < 0.05$. Statistical difference between data sets with uncoated and IgA-coated BEVs as well as PGN and co were compared by Mann-Whitney U-test as indicated; For comparisons with significant differences the exact *P* values are indicated. Source data are provided as a Source Data file.

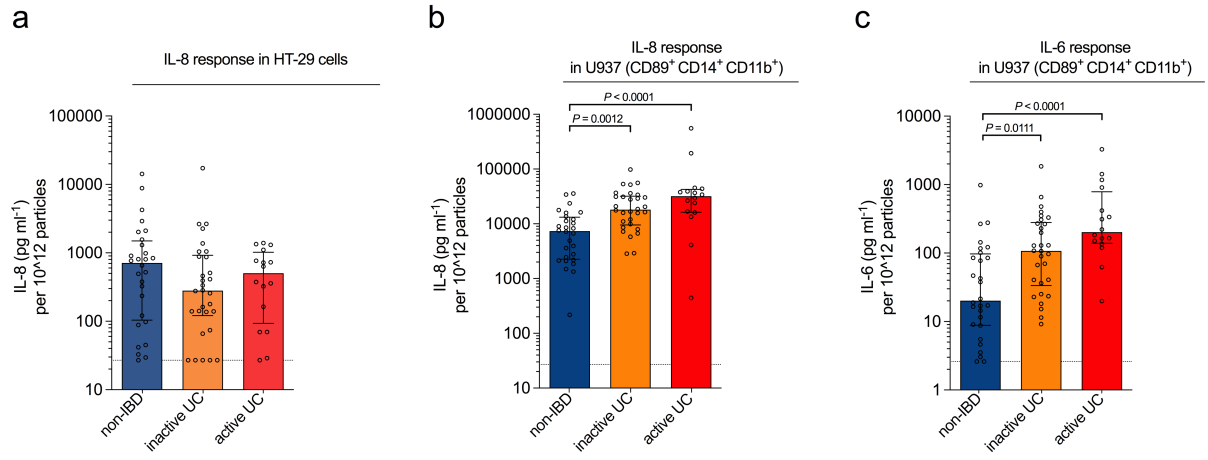

Supplementary Figure 5: Pro-inflammatory potency of EV fractions normalized to nanoparticle amount.

a, IL-8 response in HT-29 human intestinal epithelial cells exposed to extracellular vesicle (EV) fractions of the non-IBD (blue), inactive UC (yellow) and active UC (red) group normalized to the individual nanoparticle amount determined by nanoparticle tracking analysis (NTA).

b, IL-8 response in CD89$^+$CD14$^+$CD11b$^+$ U937 monocytes exposed to extracellular vesicle (EV) fractions of the non-IBD (blue), inactive UC (yellow) and active UC (red) group normalized to the individual nanoparticle amount determined by nanoparticle tracking analysis (NTA).

c, IL-6 response in CD89$^+$CD14$^+$CD11b$^+$ U937 monocytes exposed to extracellular vesicle (EV) fractions of the non-IBD (blue), inactive UC (yellow) and active UC (red) group normalized to the individual nanoparticle amount determined by nanoparticle tracking analysis (NTA).

a - c, Sample numbers are as follows: non-IBD (n=28), inactive UC (n=30) and active UC (n=16). Data is presented as median ± interquartile range. Samples with no detectable signal were set to the limit of detection, which is indicated by a dotted line. Statistical difference between the three groups was evaluated by Kruskal–Wallis with Dunn's test. Source data are provided as a Source Data file.

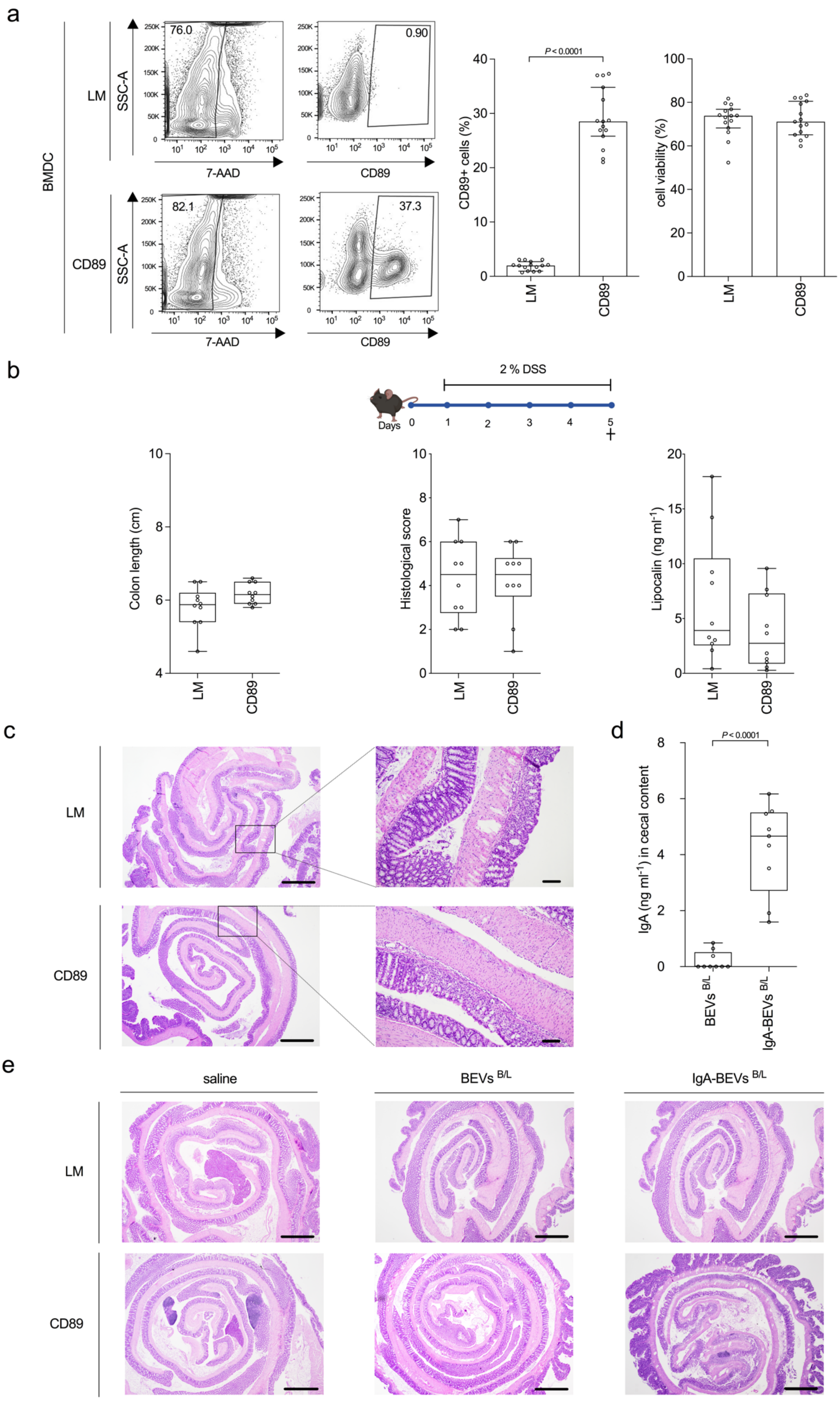
a
BMDC
LM
CD89
SSC-A
7-AAD
CD89
76.0
0.90
82.1
37.3
P < 0.0001
CD89+ cells (%)
cell viability (%)
b
2 % DSS
Days
Colon length (cm)
Histological score
Lipocalin (ng ml⁻¹)
c
d
IgA (ng ml⁻¹) in cecal content
BEVs B/L
IgA-BEVs B/L
e
saline

Supplementary Figure 6: Complimentary data associated to DSS mouse model.

a, Representative flow cytometry analysis of BMDCs prepared from CD89$^{wt/wt}$ (littermates, LM) and CD89$^{tg/wt}$ (CD89-transgenic mice, CD89) mice stained for CD89. Percentage of CD89^{+} cells of 7-AAD-negative (viable), single BMDCs derived from littermates vs CD89-transgenic mice is shown in the middle bar chart. Right bar chart shows viability of BMDCs derived from littermates vs CD89-transgenic mice determined by using the 7-AAD dye, where negative cells were considered alive. Median CD89^{+} cells of 7-AAD^{-} live, single BMDCs of littermates vs CD89-transgenic mice. Data is presented as median ± interquartile range (n =15). Statistical difference between the data sets was evaluated by two-sided Mann-Whitney U-test.

b, Shown is the timeline of the DSS treatment of CD89$^{wt/wt}$ (littermates, LM) and CD89$^{tg/wt}$ (CD89-transgenic mice, CD89) mice with harvest at day 5 (top). Bar charts (bottom) show colon length, double-blinded colon histopathology scores and lipocalin-2 levels from cecal content determined after DSS treatment harvested at day 5. Data is presented as box plot showing minimum and maximum (whiskers), interquartile range (bounds of the box), median (centre) (n = 10 mice/group). Created in BioRender. Fleischhacker, D. (2025) https://BioRender.com/1vqj75j

c, Representative histology pictures from heamotoxylin and eosin stained colons derived from CD89$^{wt/wt}$ (littermates, LM) and CD89$^{tg/wt}$ (CD89-transgenic mice, CD89) mice after DSS treatment harvested on day 5. Scale bar = 1000 µm for overview images (left) and scale bar = 100 µm for enlarged images (right). The experiments were repeated at least 10 times with a similar result.

d, Human IgA titers detected in cecal content of mice harvested at the endpoint (day 9). Mice were subjected to DSS treatement followed by oral gavage with uncoated or IgA-coated BEVs from *B. fragilis* and *L. acidophilus* (BEVs$^{B/L}$ and IgA- BEVs$^{B/L}$) according to the timeline depicted in Fig. 7a. Data is presented as box plot showing minimum and maximum (whiskers), interquartile range (bounds of the box), median (centre) (n = 9 mice/group). Statistical difference between the data sets was evaluated by two-sided Mann-Whitney U-test.

e, Shown are representative histology pictures from heamotoxylin and eosin stained colons derived from CD89$^{wt/wt}$ (littermates, LM) and CD89$^{tg/wt}$ (CD89-transgenic mice, CD89) mice after DSS treatment and oral gavage of saline, uncoated BEVs$^{B/L}$ or IgA-coated BEVs$^{B/L}$ harvested at the endpoint (day 9). Selected areas of the images are shown in main Figure 4f. Scale bar = 1000 µm. The experiments were repeated at least 10 times with a similar result.

a, b, d, Source data are provided as a Source Data file.

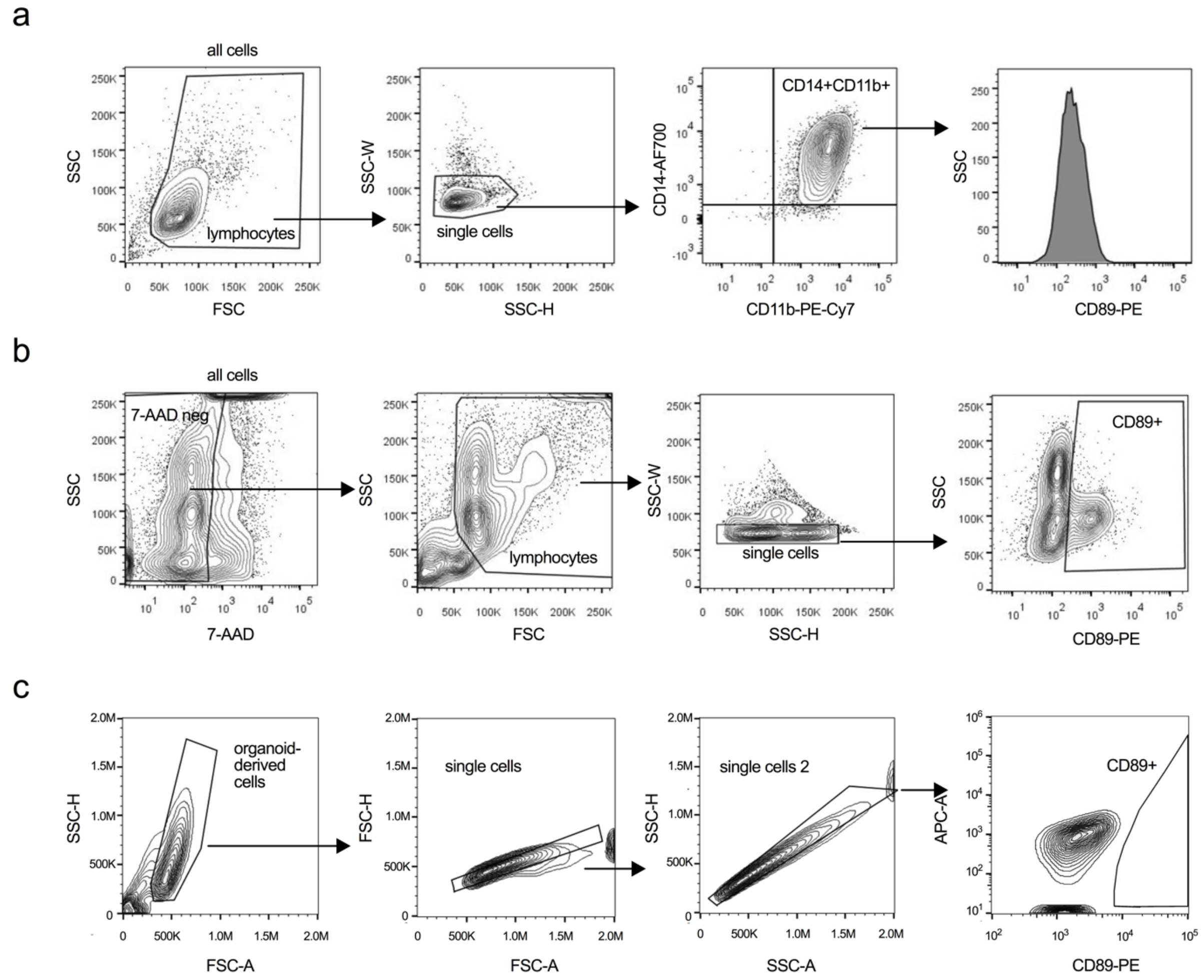

Supplementary Figure 7. Gating strategies.

a, Flow blots showing the gating strategy for U937 monocytes and CD14$^+$ monocytes (refers to Fig. 3b) isolated from human peripheral blood via magnetic sorting, respectively.

b, Flow blots showing the gating strategy for murine bone marrow derived cells (BMDC) of CD89-transgenic mice and littermates.

c, Flow blots showing the detailed gating strategy for human intestinal organoids (refers to Fig. 3a).

Supplementary Table 1. Clinical parameters of the patients (n=21) enrolled for biopsy sampling. Patients are categorized in non-IBD control group, inactive UC and active UC groups based on Mayo endoscopic subscore (inactive UC defined by a Mayo endoscopic subscore of 0 or 1; active UC defined by a Mayo endoscopic subscore of 2 or 3)[1-3]. Data is presented as absolute number/ percentage of patients or as median (minimum - maximum). Source data are provided as a Source Data file.

	**non-IBD control (n=9)**	**inactive UC (n=10)**	**active UC (n=12)**
female sex – no./ %	7/ 78%	5/ 50%	3/ 25%
age [yr]	36 (20-61)	36 (19-84)	47 (20-85)
disease duration	NA[1]	10 (4-38)	4 (0.5-21)
total Mayo score	NA	0 (0-1)	7 (4-11)
Mayo endoscopic subscore	NA	0 (0-1)	2.5 (2-3)
Montreal classification – no./ %			
E1	NA	0/ 0%	0/ 0%
E2	NA	2/ 20%	2/ 17%
E3	NA	8/ 80%	10/ 83%
Concomitant therapy – no./ %			
5-aminosalicylic acid	NA	3/ 30%	5/ 42%
steroids	NA	0/ 0%	6/ 50%
immunosuppressive drugs	NA	0/ 0%	2/ 17%
biologicals	NA	7/ 70%	5/ 42%

[1] not applicable

Supplementary Table 2: Bacterial strains and cell lines used in this study.

Strain	Characteristics	Reference
Bacteroides fragilis	Gram negative human commensal	ATCC 25285
Lactobacillus acidophilus	Gram positive human commensal	ATCC 4356
enterotoxigenic *Escherichia coli* (ETEC)	Gram negative human pathogen, H10407	[5]
**Cell line**		
HT-29	human intestinal epithelial cell line isolated from colorectal adenocarcinoma patient	ATCC HTB-38, [5]
U937	human pro-monocytic myeloid leukaemia cell line U937	ATCC CRL-1593.2, [6]

Supplementary Table 3: Antibodies used in this study.

Antibody	Dilution; final amount/ concentration	Source & order number
Alexa Fluor 488 donkey anti-rat IgG (H+L)	1.4 µg ml^{-1} (1:400)	Jackson ImmunoResearch Laboratories, 712-545-150
Alexa Fluor Plus 488-conjugated donkey anti-mouse IgG (H+L)	1:200 in NTA assays<br>&<br>1.75 µg ml^{-1} (1:800) for immunofluorescence studies	Jackson ImmunoResearch Laboratories, 715-454-150
Alexa Fluor Plus 488-conjugated goat anti-rabbit IgG (H+L)	1:200 in NTA assays	Thermo Fisher Scientific, A32731
anti-human CD11b-PE-Cy7	1:200	Biolegend, 301322
anti-human CD14-AF700	1:50	BD Bioscience, 557923
anti-human CD89-PE	1:50	BD Bioscience, 555686
avidin-HRP-conjugate	1:1000	Biolegend, 405103
biotin-conjugated anti-human IL-6 detection antibody	2.4 µg ml^{-1} (1:250)	Biolegend, 501202
biotin-conjugated anti-human IL-8 detection antibody	2.4 µg ml^{-1} (1:250)	Biolegend, 514704
Cy-3 donkey anti-rabbit IgG (H+L)	3.5 µg ml^{-1} (1:400)	Jackson ImmunoResearch Laboratories, 711-165-152
goat anti-human IgA-antibody conjugated with 20 nm gold particles	1:200 to 1:500	Cytodiagnostics, A-20-27-15
HRP-conjugated anti-rabbit IgG	1:2,000	Jackson ImmunoResearch Laboratories, JIM-111-035-003
HRP-conjugated donkey anti-human IgG	1:5,000	Biolegend, B410902
HRP-conjugated goat anti-human IgA	1:1,000	Biolegend, 411002
HRP-conjugated goat anti-mouse	1:10,000 in LTA detection assays<br>&<br>1:2,000 for dot-blot analyses	Jackson ImmunoResearch Laboratories, JIM-115-035-003
human IgG	Standard in ELISA starting at 100 ng ml^{-1}	Sigma-Aldrich, I2511
IgA from human colostrum	50 µg in IgA-coating assays	Sigma-Aldrich, I2636
IgA from human serum	50 µg in IgA-coating assays<br>&<br>Standard in ELISA starting at 100 ng ml^{-1}	Sigma-Aldrich, I4036
monoclonal mouse anti-human CD68 (clone Kp1)	1.17 µg ml^{-1} (1:300)	DakoCytomation, M0814
monoclonal rabbit anti-human CD89	0.5 µg $µl^{-1}$ (1:900)	Abcam USA, ab124717
monoclonal rat anti-mouse CD11b/ITGAM (M1/70)	1.25 µg ml^{-1} (1:80)	Cell Signaling USA, 46512

mouse anti-CD63	1:500	Thermo Fisher Scientific, 10628D
mouse anti-CD9	1:500	Thermo Fisher Scientific, 10626D
mouse anti-human CD89	1 $\mu g\ ml^{-1}$	Bio-Rad Laboratories, MCA1824
mouse anti-LTA (G43J)	1:500 in LTA detection assays<br>&<br>0.5 $\mu g\ ml^{-1}$ for IgA-LTA complex detection<br>&<br>1:50 in NTA assays<br>&<br>1:200 for TEM analyses	Thermo Fisher Scientific, MA1-7402
preadsorbed donkey anti-mouse IgG with 6 nm gold particles	1:40	abcam, ab105276
preadsorbed donkey anti-rabbit IgG conjugated with 6 nm gold particles	1:20	abcam, ab105294
purified anti-human IL-6 capture antibody	2.4 $\mu g\ ml^{-1}$	Biolegend, 501102
purified anti-human IL-8 capture antibody	2.4 $\mu g\ ml^{-1}$	Biolegend, 514602
rabbit anti-CD81	1:500	Thermo Fisher Scientific, 10630D
rabbit anti-human IgA antibody	5 µg for Dynabeads Protein G immunoprecipitation assays<br>&<br>1:100 in NTA assays	Sigma-Aldrich, SAB5600221
rabbit anti-OmpA	0.9 $\mu g\ ml^{-1}$ for for IgA-OmpA complex detection<br>&<br>1:100 for TEM analyses	Abbexa, abx110631

Supplementary Table 4: Oligonucleotides used in this study.

**Oligonucleotides**	
CD89tg P1	5′-CCAAACAGACACCCTCCTGT-3′
CD89tg P2	5′-GAGGCTTCCTTGTTCAGTGC-3′

Supplementary References

1 Sturm, A. *et al.* ECCO-ESGAR Guideline for Diagnostic Assessment in IBD Part 2: IBD scores and general principles and technical aspects. *J Crohns Colitis* **13**, 273-284, doi:10.1093/ecco-jcc/jjy114 (2019).

2 Schroeder, K. W., Tremaine, W. J. & Ilstrup, D. M. Coated oral 5-aminosalicylic acid therapy for mildly to moderately active ulcerative colitis. A randomized study. *N Engl J Med* **317**, 1625-1629, doi:10.1056/NEJM198712243172603 (1987).

3 Peyrin-Biroulet, L. *et al.* Selecting Therapeutic Targets in Inflammatory Bowel Disease (STRIDE): Determining Therapeutic Goals for Treat-to-Target. *Am J Gastroenterol* **110**, 1324-1338, doi:10.1038/ajg.2015.233 (2015).

4 Satsangi, J., Silverberg, M. S., Vermeire, S. & Colombel, J. F. The Montreal classification of inflammatory bowel disease: controversies, consensus, and implications. *Gut* **55**, 749-753, doi:10.1136/gut.2005.082909 (2006).

5 Thapa, H. B. *et al.* Characterization of the Inflammatory Response Evoked by Bacterial Membrane Vesicles in Intestinal Cells Reveals an RIPK2-Dependent Activation by Enterotoxigenic Escherichia coli Vesicles. *Microbiology spectrum* **11**, e0111523, doi:10.1128/spectrum.01115-23 (2023).

6 Jorgl, A. *et al.* Human Langerhans-cell activation triggered in vitro by conditionally expressed MKK6 is counterregulated by the downstream effector RelB. *Blood* **109**, 185-193, doi:10.1182/blood-2006-05-022954 (2007).

Study protocol

The bacterial secretome in IBD – a prospective, single-center, pilot trial

NCT04136587

Principal Investigator

Ao. Univ.-Prof. Dr. Christoph Högenauer

Universitätsklinik für Innere Medizin

Klinische Abteilung für Gastroenterologie und Hepatologie

Medizinische Universität Graz

Auenbruggerplatz 15

A-8036 Graz

0316 385 81786

christoph.hoegenauer@medunigraz.at

Subinvestigator

Priv. Doz. Dr. Dr. Andreas Blesl

Universitätsklinik für Innere Medizin

Klinische Abteilung für Gastroenterologie und Hepatologie

Medizinische Universität Graz

Auenbruggerplatz 15

A-8036 Graz

0316 385 80989

andreas.blesl@medunigraz.at

1. Background

Secreted bacterial effectors produced by the intestinal microbiota may in part be responsible for the proinflammatory effect of the fecal content in inflammatory bowel disease (IBD) patients.

2. Aim

We aimed to investigate the relevance of the bacterial secretome on inflammation in patients with ulcerative colitis and in healthy controls.

3. Inclusion and exclusion criteria

3.1. Inclusion criteria

- Healthy controls (n=20)
  - • Colonoscopy performed for the following indications: anemia, blood in stool, constipation, change in bowel habits, screening for colon cancer, follow up after polyps, weight loss
  - • Macroscopic normal colonoscopy except for diverticulosis (without any signs of inflammation), ≤ 3 polyps (except hyperplastic polyps of the colon and rectum), angiodysplasia

- Ulcerative Colitis (n=20) (two subgroups: inactive disease (10 patients) and active disease (10 patients); Definition of active UC Total Mayo score ≥ 3 and Endoscopic Mayo subscore ≥ 2; Definition of inactive UC Total Mayo score < 3 and Endoscopic Mayo subscore 0 or 1)
  - • Colonoscopy indicated by routine clinical care
  - • Established diagnosis of ulcerative colitis (also if established by the study colonoscopy)

- (Crohn´s disease; CD): n= 20 two subgroups: inactive disease (10 patients) and active disease (10 patients); Definition of active CD Harvey-Bradshaw Index - HBI ≥ 5 and / or Simple Endoscopic Score for Crohn Disease (SES-CD) ≥ 3, except SES-CD scores resulting from isolated lesions only located at the ileocolonic anastomosis consistent with a modified Rutgeerts score i2a (these patients are considered as endoscopically non active); Definition of inactive CD Harvey-Bradshaw Index (HBI) < 5 and Simple Endoscopic Score for Crohn Disease (SES-CD) < 3, except SES-CD scores resulting from isolated lesions only located at the

ileocolonic anastomosis consistent with a modified Rutgeerts score i2a (these patients are considered as endoscopically non active)

• Colonoscopy indicated by routine clinical care

• Established diagnosis of Crohn´s disease (also if established by the study colonoscopy)

- Colorectal cancer: (n=10) Definition: diagnosis of a lesion with suspicion for colorectal cancer during endoscopy which is confirmed later by histology
  • Colonoscopy indicated by routine clinical care

- Colitis/Enteritis of different origin: (n=20); Definition: diagnosis of intestinal inflammation at endoscopy or histology; E.g.: Infectious colitis /enteritis; ischemic Colitis; microscopic colitis; GVHD; NSAID colitis; Colitis of unknown cause

  • Colonoscopy indicated by routine clinical care

3.2. Exclusion criteria

- Healthy controls
  • Diagnosis of IBD or any other inflammatory condition of the small and large intestine
  • Diagnosis of irritable bowel syndrome (IBS)
  • Autoimmune disorders
  • Obesity (BMI> 30)
  • Regular intake of NSAIDs (> 2 tablets/ week) and immunosuppressants
  • Intake of antibiotics within the last 3 months
  • Intestinal infection by enteric pathogens
  • Probiotic therapy
- Patients with ulcerative colitis and Crohn´s diesease
  - Intestinal infection by enteric pathogens

4. Endpoint

Measurement of proinflammatory cytokines (f.e.: IL-8) in epithelial and dendritic tissue culture assays

5. Methods

Patients scheduled for a clinical-indicated colonoscopy at the endoscopy department of the Division of Gastroenterology and Hepatology, Medical University of Graz are eligible for the study. Patients will not receive colonoscopy for study purposes only. After obtaining informed consent, eight additional study biopsies from the sigmoid colon will be collected for study purposes additionally to routine biopsies. The sample collection and the inform consent are covered with the previous obtained ethic vote 34-460 ex 21/22 (Prospective sample collection for research on gastrointestinal diseases, Prof. Christoph Högenauer).

Fecal colonic content will be collected by suction during routine colonoscopy. From the fecal content different fractions (containing soluble bacterial fractions and membrane bound bacterial fractions) will be processed. These bacterial fractions (also termed as bacterial secretome) will be tested for proinflammatory potential in tissue culture assays including epithelial colonic cell lines and dendritic/monocytic cell lines. The main outcome parameter will be the secretion of proinflammatory cytokines from the tissue culture.

The clinical data of the included patients will be collected from the patient’s medical records via open Medocs. The assignment of the samples to the clinical data will be possible via a list of the pseudonymized samples maintained by the study director.

6. Sample size calculation

As this an explorative pilot study without sufficient pre-data, a sample size calculation is not feasible.

7. Statistics

The statistical analysis will be carried out using SPSS and R. Patient characteristics will be reported as absolute and relative frequencies for categorical data and, depending on the distribution, as median and IQR or mean and standard deviation for numerical data. Group comparisons will be made as required using the Mann-Whitney U, Kruskal-Wallis, Chi-square or Fisher exact tests. A 5% significance level will be assumed.

8. Privacy protection

All patients will be coded with a consecutive number and thus pseudonymized. The data for patient identification will only be stored in an Excel table on a computer with access restrictions at the Division of Gastroenterology and Hepatology.

9. Risk-benefit ratio

As this is a basic science project, there will be no direct benefit for the participating patients, but the study may lead to a better understanding of the diseases probably enabling new therapeutic approaches.

10. Clinical study team

Univ. FA. Dr. Lukas Binder

Andreas Posch, BSc

Andrea Streit, BSc, MsC
