# Supplementary material for: Enrichment of human IgA-coated bacterial vesicles in ulcerative colitis as a driver of inflammation
Source: Nat Commun. 2025 Apr 29;16:3995. doi: 10.1038/s41467-025-59354-5 (PMC12041585; doi:10.1038/s41467-025-59354-5)
Supplement: Supplementary file 2 — Reporting Summary [file 41467_2025_59354_MOESM2_ESM.pdf]

nature portfolio

Corresponding author(s): Stefan Schild

Last updated by author(s): 13.8.2024

Reporting Summary

Nature Portfolio wishes to improve the reproducibility of the work that we publish. This form provides structure for consistency and transparency in reporting. For further information on Nature Portfolio policies, see our Editorial Policies and the Editorial Policy Checklist.

Statistics

For all statistical analyses, confirm that the following items are present in the figure legend, table legend, main text, or Methods section.

n/a	Confirmed	
☐	☒	The exact sample size (*n*) for each experimental group/condition, given as a discrete number and unit of measurement
☐	☒	A statement on whether measurements were taken from distinct samples or whether the same sample was measured repeatedly
☐	☒	The statistical test(s) used AND whether they are one- or two-sided<br>*Only common tests should be described solely by name; describe more complex techniques in the Methods section.*
☒	☐	A description of all covariates tested
☐	☒	A description of any assumptions or corrections, such as tests of normality and adjustment for multiple comparisons
☐	☒	A full description of the statistical parameters including central tendency (e.g. means) or other basic estimates (e.g. regression coefficient) AND variation (e.g. standard deviation) or associated estimates of uncertainty (e.g. confidence intervals)
☐	☒	For null hypothesis testing, the test statistic (e.g. *F*, *t*, *r*) with confidence intervals, effect sizes, degrees of freedom and *P* value noted<br>*Give P values as exact values whenever suitable.*
☒	☐	For Bayesian analysis, information on the choice of priors and Markov chain Monte Carlo settings
☒	☐	For hierarchical and complex designs, identification of the appropriate level for tests and full reporting of outcomes
☐	☒	Estimates of effect sizes (e.g. Cohen's *d*, Pearson's *r*), indicating how they were calculated

Our web collection on statistics for biologists contains articles on many of the points above.

Software and code

Policy information about availability of computer code

Data collection	Nanoparticle tracking analysis (NTA) data was collected with a NanoSight NS300 instrument & NTA 3.4 software (Malvern Panalytical Ltd). Chemiluminescence was detected with a ChemiDoc XRS system & Quantity One software (Bio-Rad Laboratories). ELISA data was collected with a SPECTROstar nano plate reader & MARS Data Analysis Software V4.20 (BMG Labtech, Germany) . Images were recorded on the following microscopes: FEI Tecnai G2 Spirit TEM (FEI, Hillsboro, USA) & digital camera Gatan Orius 1000 (4K x 2.6k pixels), Nikon Eclipse Ni or Leica DM4000 B microscope (Leica Cambridge Ltd). Flow cytometry experiments were measured with a LRS Fortessa flow cytometer with FACSDiva 9.0.1 (BD Biosciences).
Data analysis	Data was organized and collected in Microsoft Excel (16.16.27). Graphical visualization and statistical analyses were performed in GraphPad Prism (8.4.0) and CorelDRAW 2021 (23.1.0.389). Figure 1a has been created with BioRender.com. Nanoparticle tracking analysis (NTA) data were analyzed with the NTA 3.4 software (Malvern Panalytical Ltd). Chemiluminescence Intensities were analyzed with the Quantity One software (Bio-Rad Laboratories). Flow cytometry data were analyzed usingFlowJo™ 10 (FlowJo, LLC) software.

For manuscripts utilizing custom algorithms or software that are central to the research but not yet described in published literature, software must be made available to editors and reviewers. We strongly encourage code deposition in a community repository (e.g. GitHub). See the Nature Portfolio guidelines for submitting code & software for further information.

Data

Policy information about availability of data

All manuscripts must include a data availability statement. This statement should provide the following information, where applicable:

- Accession codes, unique identifiers, or web links for publicly available datasets
- A description of any restrictions on data availability
- For clinical datasets or third party data, please ensure that the statement adheres to our policy

Whenever possible the data generated in this study are provided in the Source Data file. The clinical data is provided as pseudonymized data in the Source data file. The FACS and image data generated in this study have been deposited in the public repository https://zenodo.org under accession codes doi: 10.5281/zenodo.15019428 and doi: 10.5281/zenodo.15023687. Details on the clinical data and additional human cohort information are available under restricted access for reasons of sensitivity, access can be obtained from the corresponding author (christoph.hoegenauer@medunigraz.at) upon reasonable request within 8 weeks.

Research involving human participants, their data, or biological material

Policy information about studies with human participants or human data. See also policy information about sex, gender (identity/presentation), and sexual orientation and race, ethnicity and racism.

Reporting on sex and gender	Patients sex was self-reported by the patients. The according data are shown in Figure 1b and Table S1. For the main experimental groups the sex distribution is equal. As in UC the incidence is reported to be equal between the sexes we did not consider the sex as a special inclusion criterion for any group.
Reporting on race, ethnicity, or other socially relevant groupings	As nearly all patients included on the study were born in Austria or neighboring countries, nearly all patients were Caucasians. We therefore did not report the ethnicity in the demographic data of the patients. As UC can occur in all races, ethnicities of social groups, neither race, ethnicity of social status were an inclusion or exclusion criterion in our study.
Population characteristics	Demographic data on age sex, diagnosis and data on characterizing UC as disease activity, disease extent, disease duration and concomitant UC treatment are reported in Figure 1b and Table S1.
Recruitment	Study subjects undergoing scheduled colonoscopy at the endoscopy unit of the Division of Gastroenterology and Hepatology (Medical University of Graz) were recruited for the study if the met the inclusion and exclusion criteria reported in the material and methods section of the main manuscript. Subjects were recruited in two phases in between May and October 2019 as well as between October 2020 and September 2021. The reason the interruption in the recruitment was a lack of analytical personal in the laboratory and the first phase of COVID pandemic, were we decided to do not expose the laboratory personal to potentially infective human samples.<br>The recruitment of patients was based on the eligibility of the subject regarding inclusion and exclusion criteria as well as availability of laboratory resources for immediate samples processing. For the non-IBD group preferentially younger patients were selected to avoid any age-related bias.<br>Inclusion and exclusion criteria for the particular study groups were as follows:<br>Non-IBD control group:<br>Inclusion criteria:<br>negative history for intestinal and autoimmune diseases and colonoscopy performed for the following indications: anemia, blood in stool, constipation, change in bowel habits, screening for colon cancer, follow up after colonic polyps and weight loss; a macroscopic normal colonoscopy except for diverticulosis (without any signs of inflammation), ≤ 3 small polyps (except of hyperplastic polyps of the colon and rectum) or angiodysplasia.<br>Exclusion criteria:<br>Diagnosis of IBD or any other inflammatory condition of the small and large intestine, diagnosis of irritable bowel syndrome, acute or chronic diarrhea, autoimmune disorders, obesity (body mass index ≥ 30), regular intake of non-steroidal anti-inflammatory drugs (NSAIDs; > 2 tablets/ week) or immunosuppressants, intake of antibiotics within the last 3 months, intestinal infection by enteric pathogens and probiotic therapy;<br>UC group:<br>Inclusion criteria:<br>established or suspected (which was later confirmed) diagnosis of UC by based on the current guidelines of the European Crohn`s and Colitis Organization61;<br>Exclusion criteria:<br>Diagnosis of Crohn´s disease and infections by enteric pathogens;<br><br>Using the same assessment of UC activity as well as the same inclusion and exclusion criteria, human biopsies for the immunofluorescence staining were collected from independent patients during 2023/2024.<br>Study subjects undergoing scheduled colonoscopy at the endoscopy unit of the Division of Gastroenterology and Hepatology (Medical University of Graz) were recruited for the study if the met the inclusion and exclusion criteria reported in the material and methods section of the main manuscript. Subjects were recruited in two phases in between May and October 2019 as well as between October 2020 and September 2021. The reason the interruption in the recruitment was a lack of analytical personal in the laboratory and the first phase of COVID pandemic, were we decided to do not expose the laboratory personal to potentially infective human samples.<br>The recruitment of patients was based on the eligibility of the subject regarding inclusion and exclusion criteria as well as availability of laboratory resources for immediate samples processing. For the non-IBD group preferentially younger patients were selected to avoid any age-related bias.<br>Inclusion and exclusion criteria for the particular study groups were as follows:

Non-IBD control group:
Inclusion criteria:
negative history for intestinal and autoimmune diseases and colonoscopy performed for the following indications: anemia, blood in stool, constipation, change in bowel habits, screening for colon cancer, follow up after colonic polyps and weight loss; a macroscopic normal colonoscopy except for diverticulosis (without any signs of inflammation), ≤ 3 small polyps (except of hyperplastic polyps of the colon and rectum) or angiodysplasia.
Exclusion criteria:
Diagnosis of IBD or any other inflammatory condition of the small and large intestine, diagnosis of irritable bowel syndrome, acute or chronic diarrhea, autoimmune disorders, obesity (body mass index ≥ 30), regular intake of non-steroidal anti-inflammatory drugs (NSAIDs; > 2 tablets/ week) or immunosuppressants, intake of antibiotics within the last 3 months, intestinal infection by enteric pathogens and probiotic therapy;
UC group:
Inclusion criteria:
established or suspected (which was later confirmed) diagnosis of UC by based on the current guidelines of the European Crohn`s and Colitis Organization61;
Exclusion criteria:
Diagnosis of Crohn´s disease and infections by enteric pathogens;

Using the same assessment of UC activity as well as the same inclusion and exclusion criteria, human biopsies for the immunofluorescence staining were collected from independent patients during 2023/2024.
Study subjects undergoing scheduled colonoscopy at the endoscopy unit of the Division of Gastroenterology and Hepatology (Medical University of Graz) were recruited for the study if the met the inclusion and exclusion criteria reported in the material and methods section of the main manuscript. Subjects were recruited in two phases in between May and October 2019 as well as between October 2020 and September 2021. The reason the interruption in the recruitment was a lack of analytical personal in the laboratory and the first phase of COVID pandemic, were we decided to do not expose the laboratory personal to potentially infective human samples.
The recruitment of patients was based on the eligibility of the subject regarding inclusion and exclusion criteria as well as availability of laboratory resources for immediate samples processing. For the non-IBD group preferentially younger patients were selected to avoid any age-related bias.
Inclusion and exclusion criteria for the particular study groups were as follows:
Non-IBD control group:
Inclusion criteria:
negative history for intestinal and autoimmune diseases and colonoscopy performed for the following indications: anemia, blood in stool, constipation, change in bowel habits, screening for colon cancer, follow up after colonic polyps and weight loss; a macroscopic normal colonoscopy except for diverticulosis (without any signs of inflammation), ≤ 3 small polyps (except of hyperplastic polyps of the colon and rectum) or angiodysplasia.
Exclusion criteria:
Diagnosis of IBD or any other inflammatory condition of the small and large intestine, diagnosis of irritable bowel syndrome, acute or chronic diarrhea, autoimmune disorders, obesity (body mass index ≥ 30), regular intake of non-steroidal anti-inflammatory drugs (NSAIDs; > 2 tablets/ week) or immunosuppressants, intake of antibiotics within the last 3 months, intestinal infection by enteric pathogens and probiotic therapy;
UC group:
Inclusion criteria:
established or suspected (which was later confirmed) diagnosis of UC by based on the current guidelines of the European Crohn`s and Colitis Organization61;
Exclusion criteria:
Diagnosis of Crohn´s disease and infections by enteric pathogens;

Using the same assessment of UC activity as well as the same inclusion and exclusion criteria, human biopsies for the immunofluorescence staining were collected from independent patients during 2023/2024.

Ethics oversight

This study conformed to the principles of the Declaration of Helsinki, Good Clinical Practice Guidelines and the protocols for obtaining human samples was approved by the local ethics committee of the Medical University of Graz, (17–199 ex 05/06, 36-116 ex 23/24 & 34-460 ex 21/22 for collection of colonic luminal fluid samples and for obtaining colonic biopsies, both assigned to Christoph Högenauer). Written informed consent was obtained from all patients included in the study. Participants received no compensation. Patient samples were pseudonymized by the clinicians after harvest. The study protocol was published under clinicaltrials.gov (NCT04136587). The protocol for the establishment of human organoid cultures was approved by the ethics committee of the Medical University of Vienna (1260/2022).

Note that full information on the approval of the study protocol must also be provided in the manuscript.

Field-specific reporting

Please select the one below that is the best fit for your research. If you are not sure, read the appropriate sections before making your selection.

- [x] Life sciences
- [ ] Behavioural & social sciences
- [ ] Ecological, evolutionary & environmental sciences

For a reference copy of the document with all sections, see nature.com/documents/nr-reporting-summary-flat.pdf

Life sciences study design

All studies must disclose on these points even when the disclosure is negative.

Sample size	Patients were recruited prospectively for the individual groups. As no a priori comparison and hypothesis were made on group differences, no statistical methods were used to pre-determine sample sizes. However, our sample sizes are similar to those reported in previous publications 27-30.<br>The sample size was sufficient to demonstrate statistical significance in comparisons between the different experimental groups (non-IBD versus UC). The sample size was also suitable as demonstrated by the reproducibility in different analytical experiments.<br>Patients were recruited prospectively for the individual groups. As no a priori comparison and hypothesis were made on group differences, no statistical methods were used to pre-determine sample sizes. However, our sample sizes are similar to those reported in previous publications 27-30.<br>The sample size was sufficient to demonstrate statistical significance in comparisons between the different experimental groups (non-IBD versus UC). The sample size was also suitable as demonstrated by the reproducibility in different analytical experiments.<br>Patients were recruited prospectively for the individual groups. As no a priori comparison and hypothesis were made on group differences, no statistical methods were used to pre-determine sample sizes. However, our sample sizes are similar to those reported in previous publications 27-30.<br>The sample size was sufficient to demonstrate statistical significance in comparisons between the different experimental groups (non-IBD versus UC). The sample size was also suitable as demonstrated by the reproducibility in different analytical experiments.
Data exclusions	In total three samples were excluded (one sample of the non-IBD, inactive UC group, and active UC, respectively) as they were obtained twice from the same patient along the recruitement phase at different days and different colonoscopy procedures.
Replication	Each sample is taken from an individual human patient at a specific time point and is by nature irreproducible.
Randomization	No randomization was performed. The samples were categorized by disease (UC versus non-IBD controls) and disease activity (active UC versus inactive UC) based on current clinical guidelines.
Blinding	Blinding was not relevant in this observational study.

Behavioural & social sciences study design

All studies must disclose on these points even when the disclosure is negative.

Study description	*Briefly describe the study type including whether data are quantitative, qualitative, or mixed-methods (e.g. qualitative cross-sectional, quantitative experimental, mixed-methods case study).*
Research sample	*State the research sample (e.g. Harvard university undergraduates, villagers in rural India) and provide relevant demographic information (e.g. age, sex) and indicate whether the sample is representative. Provide a rationale for the study sample chosen. For studies involving existing datasets, please describe the dataset and source.*
Sampling strategy	*Describe the sampling procedure (e.g. random, snowball, stratified, convenience). Describe the statistical methods that were used to predetermine sample size OR if no sample-size calculation was performed, describe how sample sizes were chosen and provide a rationale for why these sample sizes are sufficient. For qualitative data, please indicate whether data saturation was considered, and what criteria were used to decide that no further sampling was needed.*
Data collection	*Provide details about the data collection procedure, including the instruments or devices used to record the data (e.g. pen and paper, computer, eye tracker, video or audio equipment) whether anyone was present besides the participant(s) and the researcher, and whether the researcher was blind to experimental condition and/or the study hypothesis during data collection.*
Timing	*Indicate the start and stop dates of data collection. If there is a gap between collection periods, state the dates for each sample cohort.*
Data exclusions	*If no data were excluded from the analyses, state so OR if data were excluded, provide the exact number of exclusions and the rationale behind them, indicating whether exclusion criteria were pre-established.*
Non-participation	*State how many participants dropped out/declined participation and the reason(s) given OR provide response rate OR state that no participants dropped out/declined participation.*
Randomization	*If participants were not allocated into experimental groups, state so OR describe how participants were allocated to groups, and if allocation was not random, describe how covariates were controlled.*

Ecological, evolutionary & environmental sciences study design

All studies must disclose on these points even when the disclosure is negative.

Study description	*Briefly describe the study. For quantitative data include treatment factors and interactions, design structure (e.g. factorial, nested, hierarchical), nature and number of experimental units and replicates.*

Research sample	*Describe the research sample (e.g. a group of tagged Passer domesticus, all Stenocereus thurberi within Organ Pipe Cactus National Monument), and provide a rationale for the sample choice. When relevant, describe the organism taxa, source, sex, age range and any manipulations. State what population the sample is meant to represent when applicable. For studies involving existing datasets, describe the data and its source.*
Sampling strategy	*Note the sampling procedure. Describe the statistical methods that were used to predetermine sample size OR if no sample-size calculation was performed, describe how sample sizes were chosen and provide a rationale for why these sample sizes are sufficient.*
Data collection	*Describe the data collection procedure, including who recorded the data and how.*
Timing and spatial scale	*Indicate the start and stop dates of data collection, noting the frequency and periodicity of sampling and providing a rationale for these choices. If there is a gap between collection periods, state the dates for each sample cohort. Specify the spatial scale from which the data are taken*
Data exclusions	*If no data were excluded from the analyses, state so OR if data were excluded, describe the exclusions and the rationale behind them, indicating whether exclusion criteria were pre-established.*
Reproducibility	*Describe the measures taken to verify the reproducibility of experimental findings. For each experiment, note whether any attempts to repeat the experiment failed OR state that all attempts to repeat the experiment were successful.*
Randomization	*Describe how samples/organisms/participants were allocated into groups. If allocation was not random, describe how covariates were controlled. If this is not relevant to your study, explain why.*
Blinding	*Describe the extent of blinding used during data acquisition and analysis. If blinding was not possible, describe why OR explain why blinding was not relevant to your study.*

Did the study involve field work? ☐ Yes ☐ No

Field work, collection and transport

Field conditions	*Describe the study conditions for field work, providing relevant parameters (e.g. temperature, rainfall).*
Location	*State the location of the sampling or experiment, providing relevant parameters (e.g. latitude and longitude, elevation, water depth).*
Access & import/export	*Describe the efforts you have made to access habitats and to collect and import/export your samples in a responsible manner and in compliance with local, national and international laws, noting any permits that were obtained (give the name of the issuing authority, the date of issue, and any identifying information).*
Disturbance	*Describe any disturbance caused by the study and how it was minimized.*

Reporting for specific materials, systems and methods

We require information from authors about some types of materials, experimental systems and methods used in many studies. Here, indicate whether each material, system or method listed is relevant to your study. If you are not sure if a list item applies to your research, read the appropriate section before selecting a response.

Materials & experimental systems

n/a	Involved in the study	
☐	☒	Antibodies
☐	☒	Eukaryotic cell lines
☒	☐	Palaeontology and archaeology
☐	☒	Animals and other organisms
☒	☐	Clinical data
☒	☐	Dual use research of concern
☒	☐	Plants

Methods

n/a	Involved in the study	
☒	☐	ChIP-seq
☐	☒	Flow cytometry
☒	☐	MRI-based neuroimaging

Antibodies

Antibodies used	Antibodies used in this study are listed in alpahtbetical order as follows: antibody name - distributor/ company - order number<br>Alexa 488 donkey anti-mouse IgG (H+L) – Jackson ImmunoResearch - 715-545-150;<br>Alexa Fluor 488 donkey anti-rat IgG (H+L) - Jackson ImmunoResearch - 712-454-150;<br>Alexa Fluor™ Plus 488- conjugated goat anti-rabbit IgG (H+L) - Thermo Fisher Scientific - A32731;<br>anti-CD89 antibody (MIP8a) - Bio-Rad - MCA1824;

Anti-human CD11b-PE-Cy7 (ICRF44) - Biolegend – 301322;
Anti-human CD14-AF700 (M5E2) - BD Bioscience – 557923;
anti-human CD89-PE (A59) - BD Biosciences – 555686;
anti-human IgA-antibody conjugated with 20 nm gold particles - Cytodiagnostics - A-20-27-15;
anti-rabbit IgG conjugated with 6 nm gold particles -Abcam- ab105294;
anti-mouse IgG with 6 nm gold particles -Abcam- ab105276;
CD81 Monoclonal Antibody - Invitrogen - 10630D;
CD9 Monoclonal Antibody - Invitrogen - 10626D;
CD63 Monoclonal Antibody - Invitrogen - 10628D
Cy-3 donkey anti-rabbit IgG (H+L) - Jackson ImmunoResearch - 711-165-152;
HRP conjugated Goat anti-mouse - Jackson ImmunoResearch Laboratories - JIM-115-035-003;
HRP conjugated Goat anti-rabbit - Jackson ImmunoResearch - JIM-111-035-003;
HRP Donkey anti-human IgG - Biolegend - B410902;
HRP Goat anti-human IgA - Biolegend – 411002;
HRP-conjugated goat anti-mouse - Jackson ImmunoResearch - 115-035-003;
LTA Monoclonal Antibody (G43J) - Thermo Fischer Scientific - MA1-7402;
anti-OmpA – Abbexa BV - abx110631;
mouse anti-human CD68 (Kp1) - Dako - M 0814;
Purified anti-human IL-6 - Biolegend - B501102;
Purified anti-human IL-8 - Biolegend – 514602;
Rabbit anti-human CD89 (EPR4622(2)) - Abcam - ab 124717;
rabbit anti-human IgA antibody - Sigma-Aldrich - SAB5600221;
rat anti-mouse CD11b/ITGAM (M1/70) - Cell Signaling – 46512;

Validation

All antibodies are commercially available and validated for the use of immunofluorescence, flow cytometry analyses, immunoblot or ELISA. Respective data are available on the individual manufacturers websites.

Eukaryotic cell lines

Policy information about cell lines and Sex and Gender in Research

Cell line source(s)

HT-29 (source: ATCC® HTB-38) and U937 (source: ATCC CRL-1593.2, doi:10.1182/blood-2006-05-022954)

Authentication

The cell lines were not authenticated after receipt

Mycoplasma contamination

Cell lines were not recently tested for mycoplasma contamination

Commonly misidentified lines
(See ICLAC register)

No commonly misidentified cell line was used.

Palaeontology and Archaeology

Specimen provenance

Provide provenance information for specimens and describe permits that were obtained for the work (including the name of the issuing authority, the date of issue, and any identifying information). Permits should encompass collection and, where applicable, export.

Specimen deposition

Indicate where the specimens have been deposited to permit free access by other researchers.

Dating methods

If new dates are provided, describe how they were obtained (e.g. collection, storage, sample pretreatment and measurement), where they were obtained (i.e. lab name), the calibration program and the protocol for quality assurance OR state that no new dates are provided.

☐ Tick this box to confirm that the raw and calibrated dates are available in the paper or in Supplementary Information.

Ethics oversight

Identify the organization(s) that approved or provided guidance on the study protocol, OR state that no ethical approval or guidance was required and explain why not.

Note that full information on the approval of the study protocol must also be provided in the manuscript.

Animals and other research organisms

Policy information about studies involving animals; ARRIVE guidelines recommended for reporting animal research, and Sex and Gender in Research

Laboratory animals

Myeloid-specific CD89-expressing mice were generated by crossing CD89tg/wt (source: CSL Limited, Australia) and C57BL/6 LyzMcre/cre Tg mice (source: Jackson Laboratories) to excise the loxP-flanked mCherry cassette in vivo as previously reported (doi:10.1038/s41385-019-0167-z). Excision of the mCherry cassette results CD89 expression in the myeloid cell lineage under a CMV promoter in 50% of the offspring (CD89tg/wt/LyzMcre/wt; CD89+). Littermates (CD89wt/wt/LyzMcre/wt; LM) not expressing CD89 served as

	CD89-negative controls.
Wild animals	The study did not involve wild animals
Reporting on sex	Only 7 to 8 week old female mice were used in this study.
Field-collected samples	The study did not involve samples collected from the field.
Ethics oversight	All mouse studies were performed in accordance with the Commission for Animal Experiments of the Austrian Ministry of Science (animal protocol: GZ BMWFW-39/8/75 ex 2020/21) and the local Animal Welfare Committee of the University of Graz (Head: Kathrin Zierler).

Note that full information on the approval of the study protocol must also be provided in the manuscript.

Clinical data

Policy information about clinical studies

All manuscripts should comply with the ICMJE guidelines for publication of clinical research and a completed CONSORT checklist must be included with all submissions.

Clinical trial registration	*Provide the trial registration number from ClinicalTrials.gov or an equivalent agency.*
Study protocol	*Note where the full trial protocol can be accessed OR if not available, explain why.*
Data collection	*Describe the settings and locales of data collection, noting the time periods of recruitment and data collection.*
Outcomes	*Describe how you pre-defined primary and secondary outcome measures and how you assessed these measures.*

Dual use research of concern

Policy information about dual use research of concern

Hazards

Could the accidental, deliberate or reckless misuse of agents or technologies generated in the work, or the application of information presented in the manuscript, pose a threat to:

No	Yes	
x	☐	Public health
x	☐	National security
x	☐	Crops and/or livestock
x	☐	Ecosystems
x	☐	Any other significant area

Experiments of concern

Does the work involve any of these experiments of concern:

No	Yes	
x	☐	Demonstrate how to render a vaccine ineffective
x	☐	Confer resistance to therapeutically useful antibiotics or antiviral agents
x	☐	Enhance the virulence of a pathogen or render a nonpathogen virulent
x	☐	Increase transmissibility of a pathogen
x	☐	Alter the host range of a pathogen
x	☐	Enable evasion of diagnostic/detection modalities
x	☐	Enable the weaponization of a biological agent or toxin
x	☐	Any other potentially harmful combination of experiments and agents

Plants

Seed stocks

Report on the source of all seed stocks or other plant material used. If applicable, state the seed stock centre and catalogue number. If plant specimens were collected from the field, describe the collection location, date and sampling procedures.

Novel plant genotypes

Describe the methods by which all novel plant genotypes were produced. This includes those generated by transgenic approaches, gene editing, chemical/radiation-based mutagenesis and hybridization. For transgenic lines, describe the transformation method, the number of independent lines analyzed and the generation upon which experiments were performed. For gene-edited lines, describe the editor used, the endogenous sequence targeted for editing, the targeting guide RNA sequence (if applicable) and how the editor was applied.

Authentication

Describe any authentication procedures for each seed stock used or novel genotype generated. Describe any experiments used to assess the effect of a mutation and, where applicable, how potential secondary effects (e.g. second site T-DNA insertions, mosiacism, off-target gene editing) were examined.

ChIP-seq

Data deposition

☐ Confirm that both raw and final processed data have been deposited in a public database such as GEO.

☐ Confirm that you have deposited or provided access to graph files (e.g. BED files) for the called peaks.

Data access links
May remain private before publication.

For "Initial submission" or "Revised version" documents, provide reviewer access links. For your "Final submission" document, provide a link to the deposited data.

Files in database submission

Provide a list of all files available in the database submission.

Genome browser session
(e.g. UCSC)

Provide a link to an anonymized genome browser session for "Initial submission" and "Revised version" documents only, to enable peer review. Write "no longer applicable" for "Final submission" documents.

Methodology

Replicates

Describe the experimental replicates, specifying number, type and replicate agreement.

Sequencing depth

Describe the sequencing depth for each experiment, providing the total number of reads, uniquely mapped reads, length of reads and whether they were paired- or single-end.

Antibodies

Describe the antibodies used for the ChIP-seq experiments; as applicable, provide supplier name, catalog number, clone name, and lot number.

Peak calling parameters

Specify the command line program and parameters used for read mapping and peak calling, including the ChIP, control and index files used.

Data quality

Describe the methods used to ensure data quality in full detail, including how many peaks are at FDR 5% and above 5-fold enrichment.

Software

Describe the software used to collect and analyze the ChIP-seq data. For custom code that has been deposited into a community repository, provide accession details.

Flow Cytometry

Plots

Confirm that:

☐ The axis labels state the marker and fluorochrome used (e.g. CD4-FITC).

☒ The axis scales are clearly visible. Include numbers along axes only for bottom left plot of group (a 'group' is an analysis of identical markers).

☒ All plots are contour plots with outliers or pseudocolor plots.

☐ A numerical value for number of cells or percentage (with statistics) is provided.

Methodology

Sample preparation

CD14+ monocytes were isolated from buffy coat preparations obtained from the Department for Blood Serology and Transfusion Medicine, Medical Hospital Graz. CD14+ cells were positively selected from MNC fractions (prepared with Lymphoprep (Axis Shield)) with magnetic beads using the Human CD14 MicroBeads isolation kit (Miltenyi) according to the manufacturer’s protocol.
BMDCs were isolated from mouse bone-marrow precursors. In brief, femur and tibiae were removed from 7–8-week-old mice (CD89 and WT). Bone marrow cells were flushed out with RPMI 1640 medium (Sigma-Aldrich) supplemented with 1% penicillin/streptomycin and 10% FBS, purified through a 100 µm cell strainer (Corning) and incubated at 37 °C and 5% CO2 in

a humidified atmosphere for 24 h. On the second day, 2 x 105 BMDCs were seeded in 24-well tissue culture plates in presence of IgA-coated and uncoated bacterial MVs resuspended in RPMI 1640 medium. Mock-treated cells cultivated in growth medium served as controls. After incubation for 16 h the cells were harvested and washed with PBS.
U937 cells were maintained at 37°C and 5% CO2 in a humidified atmosphere and cultivated RPMI 1640 growth medium (Sigma-Aldrich) supplemented with 10% FBS and 1% penicillin/streptomycin (Gibco). 2.5 x 105 cells/ml were differentiated to CD89+CD14+CD11b+ monocytes by addition of vitamin D3 (100 nM, 1α,25-Dihydroxyvitamin D3, Sigma-Aldrich) and TGF-β1 (0.5 ng/ml, rhTGF-β1, R&D Systems) for 48 h. Untreated cells and cells treated with an equivalent volume of EtOH were used as control and solvent control respectively. U937 cells were collected, washed and resuspended in PBS.
In case of organoids, a single cell suspension of TrypLE dissociated organoid cultures was stained for 20 min at 4°C with anti - human CD89-PE (BD Bioscience, 1:50). Data were then acquired on a CytoFlex machine and subsequently analyzed using FlowJoTM software at the CeMM, Vienna.

Instrument

LRS Fortessa flow cytometer (BD Biosciences) or CytoFlex machine

Software

Data were recorded using FACSDiva 9.0.1 and analyzed using FlowJo™ 10 (FlowJo, LLC) software.

Cell population abundance

Purity of magnetically sorted CD14+ monocytes from peripheral blood is on average above 95%.
Above 90% of the U937 cells treated with VD3 and TGF-β1 differentiate into CD14+CD11b+CD89+ positive monocytes.
Isolated and treated BMDCs negative for 7-AAD staining were considered alive (on average 72% 7-AAD- cells, CD89 TG mice: 59.9-83.3% and LM mice: 52,3-81,7%). CD89+ population within the alive cells was on average 29.2% (CD89 TG mice, ranging between 21.0-37.3%) vs. 1,9% (LM mice, ranging between 0.7-3.1%).

Gating strategy

CD14 & U937: lymphocytes according to FSC vs. SSC > SSC-H vs. SSC-W singlets > CD14+CD11b+ > CD89+
BDMCs: 7-AAD negative alive cells > lymphocytes according to FSC vs. SSC > SSC-H vs. SSC-W singlets > CD89+
organoids: living cells according to FSC vs. SSC > FSC-H vs. FSC-A singlets > CD89+

☒ Tick this box to confirm that a figure exemplifying the gating strategy is provided in the Supplementary Information.

Magnetic resonance imaging

Experimental design

Design type

Indicate task or resting state; event-related or block design.

Design specifications

Specify the number of blocks, trials or experimental units per session and/or subject, and specify the length of each trial or block (if trials are blocked) and interval between trials.

Behavioral performance measures

State number and/or type of variables recorded (e.g. correct button press, response time) and what statistics were used to establish that the subjects were performing the task as expected (e.g. mean, range, and/or standard deviation across subjects).

Acquisition

Imaging type(s)

Specify: functional, structural, diffusion, perfusion.

Field strength

Specify in Tesla

Sequence & imaging parameters

Specify the pulse sequence type (gradient echo, spin echo, etc.), imaging type (EPI, spiral, etc.), field of view, matrix size, slice thickness, orientation and TE/TR/flip angle.

Area of acquisition

State whether a whole brain scan was used OR define the area of acquisition, describing how the region was determined.

Diffusion MRI ☐ Used ☐ Not used

Preprocessing

Preprocessing software

Provide detail on software version and revision number and on specific parameters (model/functions, brain extraction, segmentation, smoothing kernel size, etc.).

Normalization

If data were normalized/standardized, describe the approach(es): specify linear or non-linear and define image types used for transformation OR indicate that data were not normalized and explain rationale for lack of normalization.

Normalization template

Describe the template used for normalization/transformation, specifying subject space or group standardized space (e.g. original Talairach, MNI305, ICBM152) OR indicate that the data were not normalized.

Noise and artifact removal

Describe your procedure(s) for artifact and structured noise removal, specifying motion parameters, tissue signals and physiological signals (heart rate, respiration).

Volume censoring

Define your software and/or method and criteria for volume censoring, and state the extent of such censoring.

Statistical modeling & inference

Model type and settings: *Specify type (mass univariate, multivariate, RSA, predictive, etc.) and describe essential details of the model at the first and second levels (e.g. fixed, random or mixed effects; drift or auto-correlation).*

Effect(s) tested: *Define precise effect in terms of the task or stimulus conditions instead of psychological concepts and indicate whether ANOVA or factorial designs were used.*

Specify type of analysis: ☐ Whole brain ☐ ROI-based ☐ Both

Statistic type for inference: *Specify voxel-wise or cluster-wise and report all relevant parameters for cluster-wise methods.*

(See Eklund et al. 2016)

Correction: *Describe the type of correction and how it is obtained for multiple comparisons (e.g. FWE, FDR, permutation or Monte Carlo).*

Models & analysis

n/a	Involved in the study
☐	☐ Functional and/or effective connectivity
☐	☐ Graph analysis
☐	☐ Multivariate modeling or predictive analysis

Functional and/or effective connectivity: *Report the measures of dependence used and the model details (e.g. Pearson correlation, partial correlation, mutual information).*

Graph analysis: *Report the dependent variable and connectivity measure, specifying weighted graph or binarized graph, subject- or group-level, and the global and/or node summaries used (e.g. clustering coefficient, efficiency, etc.).*

Multivariate modeling and predictive analysis: *Specify independent variables, features extraction and dimension reduction, model, training and evaluation metrics.*
